# Leptin: A Heavyweight Player in Obesity-Related Cancers

**DOI:** 10.3390/biom13071084

**Published:** 2023-07-06

**Authors:** Amanda Caruso, Luca Gelsomino, Salvatore Panza, Felice Maria Accattatis, Giuseppina Daniela Naimo, Ines Barone, Cinzia Giordano, Stefania Catalano, Sebastiano Andò

**Affiliations:** 1 Department of Pharmacy, Health and Nutritional Sciences, Via P Bucci, University of Calabria, Arcavacata di Rende (CS), 87036 Cosenza, Italy; amanda.caruso@unical.it (A.C.); luca.gelsomino@unical.it (L.G.); salvatore.panza@unical.it (S.P.); felice.accattatis@gmail.com (F.M.A.); giuseppinadaniela.naimo@unical.it (G.D.N.); ines.barone@unical.it (I.B.); cinzia.giordano@unical.it (C.G.); stefania.catalano@unical.it (S.C.); 2 Centro Sanitario, Via P. Bucci, University of Calabria, Arcavacata di Rende (CS), 87036 Cosenza, Italy

**Keywords:** obesity, leptin, cancers, colorectal, lung, glioma, breast cancer, cervical, corpus uteri, ovarian

## Abstract

Obesity, defined as the abnormal or excessive expansion of white adipose tissue, has reached pandemic proportions and is recognized as an important health concern since it is a common root for several comorbidities, including malignancies. Indeed, the current knowledge of the white adipose tissue, which shifts its role from an energy storage tissue to an important endocrine and metabolic organ, has opened up new avenues for the discovery of obesity’s effects on tumor biology. In this review, we will report the epidemiological studies concerning the strong impact of obesity in several types of cancer and describe the mechanisms underlying the heterotypic signals between cancer cell lines and adipocytes, with particular emphasis on inflammation, the insulin/IGF-1 axis, and adipokines. Among the adipokines, we will further describe the in vitro, in vivo, and clinical data concerning the role of leptin, recognized as one of the most important mediators of obesity-associated cancers. In fact, leptin physiologically regulates energy metabolism, appetite, and reproduction, and several studies have also described the role of leptin in affecting cancer development and progression. Finally, we will summarize the newest pharmacological strategies aimed at mitigating the protumorigenic effects of leptin, underlining their mechanisms of action.

## 1. Introduction

Obesity is a systemic disease characterized by an excessive and abnormal accumulation of adipose tissue in the body and defined by body mass index (BMI), as stated by the World Health Organization (WHO) and the National Institutes of Health (NIH). It has been widely described that weight gain is associated with an imbalance between energy consumption and energy intake, and a value greater than 30 kg/m^2^ of BMI, calculated by using the formula weight (kg)/height (m^2^), refers to an obese individual (30.0–34.9, grade I; 35.0–39.9, grade II; and ≥40, grade III) [1]. As reported in the latest findings by the WHO, obesity has tripled worldwide since 1975 and its prevalence is higher in high-income countries versus low-income countries. Nevertheless, the number of obese people is also increasing in low-income countries, particularly in urban settings. In 2016, more than 1.9 billion adults were overweight and of these over 650 million were obese, while 39 million children under the age of 5 were overweight or obese in 2020, indicating that obesity has reached pandemic proportion and is recognized as a major health concern [2]. Moreover, besides its many health consequences, mainly related to cardiovascular diseases, diabetes, and musculoskeletal disorders, epidemiological evidence recognizes obesity as a risk factor for numerous malignancies. In fact, excess body weight is associated with tumor incidence, morbidity, and mortality [3,4]. First, we report the latest epidemiological data, tightly linking obesity and several type of cancers. Then, we will describe the molecular mechanisms underlying the obesity–cancer link, including inflammation, estrogens, insulin signaling, and adipokines. With a specific focus on adipokines, we will analyze the role of leptin, whose circulating levels proportionally increase with body fat mass. Leptin, mainly secreted by adipose tissue, is known to regulate several physiological processes, including energy metabolism, reproduction, appetite control, and immunity, but its direct biological effects on tumor development and progression have also been reported. Moreover, different studies have found that leptin circulating levels and leptin receptors are overexpressed in multiple types of cancer, resulting in a poor prognostic significance value. Thus, in this review, we will highlight in vitro, in vivo, and clinical studies concerning the role of leptin in obesity-related cancers. Finally, we will discuss the translational impact of these findings by evidencing the different mechanisms of action of drugs that are able to counteract the leptin-mediated effects and which may overcome the interplay between obesity and cancer.

## 2. The Epidemiological Association between Obesity and Cancer

**Figure 1:**
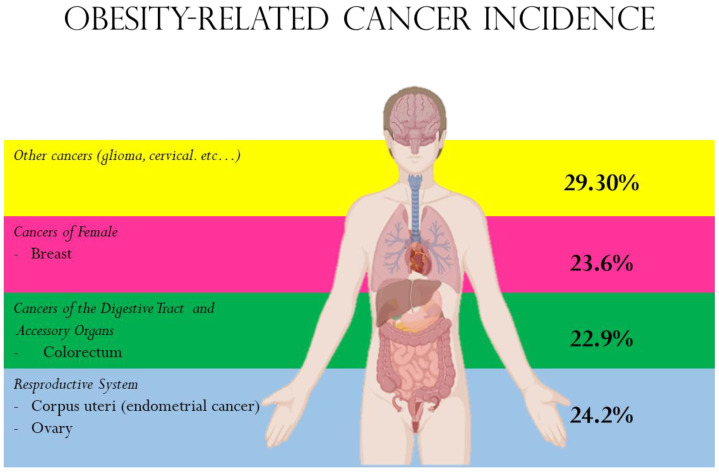
Obesity-related cancers and their relative incidence (source: https://gco.iarc.fr/causes/obesity/tools-pie, accessed on May 2023).

In 2016, The International Agency for Research on Cancer (IARC) reported that weight control is important for reducing cancer risk. Indeed, evidence reviewed by the IARC, derived from epidemiologic studies, animal models, and mechanistic studies, supports the conclusion that obesity is related to different types of cancer in terms of risk and mortality (Figure 1). Here, we will review the latest epidemiological studies on cancers that are strongly correlated with obesity (i.e., colorectal, post-menopausal breast, corpus uteri, and ovarian) and some cancers where this association is weak (i.e., lung, glioma, cervical) [5,6].

### 2.1. Colorectal Cancer

Colorectal cancer (CRC) is the third most commonly diagnosed cancer worldwide and represents the second fastest-increasing cause of cancer death (9.4% of the total cancer deaths), with increasing incidence in countries with an elevated Human Development Index (HDI) compared to those with a lower HDI [7]. Different risk factors have been associated with CRC development, among which it has been reported that obesity may affect CRC risk [8]. In a meta-analysis of 19 prospective cohort studies, derived from 18 different publications with a total of 12,837 CRC cases, it has been demonstrated that abdominal obesity, measured by the most common methods such as waist circumference (WC) and waist-to-hip ratio (WHR), influences the development of CRC without any difference between men and women and geographic region. In particular, greater WC and WHR have been associated with high relative risks (RRs) with a confidence interval (CI) of 95% of CRC for total colorectal cancer (WC: RR 1.42, 95% CI 1.30, 1.55; WHR: RR 1.39, 95% CI 1.25, 1.53), colon cancer (WC: RR 1.53, 95% CI 1.36, 1.72; WHR: 1.39, 95% CI 1.18, 1.63), and rectal cancer (WC: RR 1.20, 95% CI 1.03, 1.39; WHR: RR 1.22, 95% CI 1.05, 1.42) [9]. Moreover, it has also been reported that obese patients, according to their BMI, affected by CRC showed an increased risk of all-cause mortality (RR 1.14; 95% CI 1.07–1.21), cancer-specific mortality (RR 1.14; 95% CI 1.05–1.24), disease recurrence (RR 1.07; 95% CI 1.02–1.13), and worse disease-free survival compared to patients with a normal weight (RR 1.07; 95% CI 1.01–1.13) [10]. In another meta-analysis comprising 12 observational studies and analyzing a total of 16,151 CRC cases, it has been evidenced that even moderate weight gain is correlated with an increased risk of CRC development, and this association was more evident in men than in women. In detail, the RR was 1.22 (CI 95%: 1.14–1.30) for high body weight gain, considering 15.2 kg as the midpoint compared to stable weight. Interestingly, each 5 kg weight gain increased the risk of CRC (4%, 95% CI: 2%–5%) [11]. In line with these findings, it has been demonstrated that body fat may also contribute to the development of CRC in subjects younger than 30 years old with a high BMI. This association was not evident in rectal cancer. The meta-analysis including 15 observational studies (13 cohort studies and two case-control studies) reported that each increment of 5 kg/m^2^ in BMI was significantly associated with a 13% (RR 1.13, 95% CI 1.08, 1.19) higher risk of colorectal cancer overall, 17% in men (RR 1.17, 95% CI 1.09, 1.25) and 8% (RR 1.08, 95% CI 1.04, 1.11) in women, respectively [12]. This can be reinforced by the evidence that circulating levels of leptin, the predominant hormone secreted by adipocytes, were significantly higher in tumor patients compared to normal subjects [13,14,15].

### 2.2. Lung Cancer

Lung cancer is the principal cause of cancer incidence and death in both men and women, accounting for 2,206,771 new cases and 1,796,144 deaths in 2020 [7]. Cigarette smoking is the leading risk factor for lung cancer (responsible for almost 80% of all lung cancer mortality), but physical activity and body weight may also be responsible for lung cancer development [16]. Although different studies reported that obesity lowers lung cancer risk [17,18], this association still remains controversial, mainly owing to the effects of smoking on body weight as well as preclinical weight loss and socioeconomic status [19,20]. Recently, a study avoiding any bias by analyzing several nested case-control studies, classifying patients as current, former, or lifelong non-smokers demonstrated that there is a negative association between lung cancer risk and obesity. The authors, by investigating a total of 12,643 subjects, 4172 lung cancer cases, and 8471 controls, aged 35 to 74 years, from four cohort studies in the USA, Europe, China, and Singapore, found a statistical association between BMI and lung cancer risk both in current smokers (Odd Ratio, OR for overweight group: 0.79, 95% CI: 0.68–0.92, and obese group: 0.75, 95% CI: 0.60–0.93), former smokers (overweight group: 0.70, 95% CI: 0.53–0.93, and obese group: 0.55, 95% CI: 0.37–0.80), and lifelong non-smokers (overweight group: 0.77, 95% CI: 0.59–0.99, and obese group: 0.71, 95% CI: 0.44–1.14) [21]. Interestingly, levels of leptin, a major obesity-related adipokine, have been documented to positively correlate with non-small cell lung cancer (NSCLC) [22,23,24,25]. On the contrary, it has also been demonstrated that central or visceral adiposity, measured by visceral fat index (VFI), is associated with decreased recurrence-free and overall survival (OS) in lung cancer patients. Noteworthy is the use of VFI estimated by computed tomography images to overcome the limitations of the use of BMI for determining the adiposity [26]. Importantly, since lung cancer is rare in lifelong non-smokers, the data concerning the association between obesity and lung cancer incidence in these patients are still inconsistent. However, a study conducted by Zhu and Zhang in 2018 demonstrated that a higher BMI is associated with a lower risk of lung cancer in lifelong non-smokers, mainly in women [27].

### 2.3. Glioma

Intracranial gliomas refer to the most common primary central nervous system (CNS) tumors, such as glioblastomas (GBM), diffuse astrocytoma, and oligodendrogliomas, and they account for 70% of all brain malignancies [28]. Although many risk factors have been identified, the etiology of glioma requires further investigation. Studies suggest that there is no linear association between gliomas and obesity [29,30]. Indeed, in a large prospective cohort study involving 1.8 million Norwegian women and men, in which 4382 gliomas were identified, it has been reported that changes in height were positively associated with risk for gliomas (HR per 10 cm increase: 1.24; 95% [CI], 1.17–1.31), while BMI did not affect the risk of gliomas [31]. Other research groups have reported a possible link between an increase in body weight and the development of malignant brain tumors [32,33]. Ahn et al., based on a nationwide population-based cohort study of Koreans, involving 6,833,744 patients with documented 4771 glioma cases, found that obesity and abdominal obesity may contribute to glioma risk. For instance, a positive correlation between leptin levels and glioma was found [34]. This association between obesity/waist circumference and glioma was stronger in women than in men. Particularly, the HR for individuals with a BMI higher than 25.0 kg/m^2^ was 1.08, CI 1.02–1.15, and, with a WC higher than 90 cm for males and 85 cm for females, was HR 1.16, CI 1.09–1.24, compared to the control counterpart [32]. Another meta-analysis showed that obesity might emerge as a risk factor for all brain tumors, and, in particular, this association was more evident among women with glioma, wherein the pooled RR was 1.17, 95% CI: 1.03–1.32, while no statistically significant risk has been observed in men [33].

### 2.4. Postmenopausal Breast Cancer

Breast cancer is the most common malignancy in women, representing the fifth leading cause of cancer-associated deaths worldwide with 685,000 deaths [7]. Different epidemiological studies have reported a strong relationship between obesity and breast cancer, mainly demonstrating that the excess of adiposity profoundly increases the incidence and mortality of breast cancer patients. The association between obesity and breast cancer is also correlated with menopausal status, specific life stages, and disease subtypes. Particularly, in 2017, Gershuni et al., enrolling 848 women divided into three different groups according to their BMI (normal, overweight, and obese), demonstrated that overweight and obese status were associated with hormone receptor (HR)-positive and Human Epidermal Growth Factor 2 (HER2)-negative breast cancer (58.1%) with a more aggressive phenotype, such as triple-negative breast cancer (TNBC, 14%). On the other hand, women with a normal weight were mainly affected by HER2-positive breast cancer [35]. Moreover, another study evaluated the association between obesity and breast tumor risk, analyzing the women enrolled in the multinational European Prospective Investigation into Cancer and Nutrition (EPIC) prospective study (n = 27,012) in relation to tumor subtypes according to the expression of clinically used markers (Estrogen receptor, ER, Progesterone Receptor, PR, HER2, Ki67, Bcl-2 and p53). The results obtained clearly showed that increased BMI is related to low aggressive breast cancer subtypes, mainly expressing the ER and PR receptor in postmenopausal women [36]. Recently, a large dose–response meta-analysis on 12 prospective cohort studies comprising 22,728,674 women showed a potential linear link between BMI and breast cancer risk. In particular, it has been demonstrated that a 5 kg/m^2^ increase in BMI was related to a 2% increase in breast cancer risk, with an estimated summary relative risk (SRR) of 1.02 (95% CI: 1.01–1.04, *p* < 0.001) in postmenopausal women, while a higher BMI may represent a protective factor for breast cancer risk in premenopausal women, with a calculated SRR of 0.98 (95% CI: 0.96–0.99, *p* < 0.001) [37]. A similar positive non-linear association between BMI and breast cancer risk in postmenopausal women was also reported in another dose-response meta-analysis, with 3,318,796 participants from 31 articles. The authors showed that the RRs were 1.33 (95% CI: 1.20–1.48) and stated an increase of 3.4% for breast cancer risk for every 1 kg/m^2^ increment in BMI in postmenopausal women [38]. Noteworthy, the over-expression of leptin, a major player of obesity-related malignancies, has been found in high-grade breast cancer with poor prognosis [39,40,41,42,43,44].

### 2.5. Gynecologic Cancers (Cervical and Ovarian Cancers, and Cancer of the Corpus Uteri)

#### 2.5.1. Cervical Cancer

In 2020, an estimated 604,000 new cases and 342,000 deaths due to cervical cancer were reported, making it the fourth most frequently diagnosed cancer and the fourth leading cause of cancer death in women worldwide [7]. Besides the well-recognized risk factors that contribute to the development of cervical cancer, such as human papillomavirus (HPV) infection, other cofactors may also influence the biology of this neoplasia. In particular, obesity and lifestyle may contribute to the risk of cervical carcinogenesis. In 2018, a retrospective cohort study carried out by Kaiser Permanente Northern California (KPNC), enrolling more than 900,000 women, demonstrated that overweight and obese conditions may increase the risk of cervical cancer. Specifically, the authors reported that 5-year cumulative risk (CR) of cancer for obese women was 0.083% (95% CI), representing the highest one, since the CR for overweight and normal/underweight women was 0.071% and 0.056% (95% CI), respectively. However, a lower risk of cervical pre-cancer with increasing body mass index has also been reported, probably because cervical cancer may be misdiagnosed in overweight and obese patients [45]. Another work reported the importance of maintaining an appropriate BMI along with regular physical activity as a preventive strategy against cervical cancer. In fact, Lee and colleagues assessed that BMI positively affects the risk of cervical cancer, since for 1125 women enrolled in the study, they found that the multivariate ORs were 1.25 and 1.70 (95% CI) for overweight and obese patients, respectively, in comparison with those with a normal BMI [46]. Recently, a similar trend between obesity and cervical cancer has also been demonstrated in a large population-based cohort study in Korean women. Independently of menopausal status, class II obesity significantly increased the risk of cervical cancer. In particular, the estimated adjusted hazard ratios (aHRs) were 1.27 (95% CI) for the pre-menopausal group and 1.18 (95% CI) for the post-menopausal group [47]. The latter findings can be reinforced by the evidence that leptin was positively correlated with a higher grade of cervical carcinoma [48].

#### 2.5.2. Ovarian Cancer

Ovarian cancer, divided into serous, clear cell, endometrioid, and mucinous subtypes, represents the eighth cancer type among women in terms of incidence, with 313,959 new diagnosed cases and 202,252 deaths worldwide [7]. A two-sample mendelian randomization study estimating both BMI and body composition, assessed by bioelectrical impedance analysis, such as the trunk and arm fat ratio (TFR and AFR), showed that visceral adiposity may contribute to the risk of ovarian cancer, mainly clear cell and endometrioid cancer subtypes. In fact, compared to BMI, OR was 1.34 (95% CI: [1.11, 1.62]), AFR was 1.33 (95% CI: [1.05, 1.69]), and TFR was positively 1.10 (95% CI: [1.01, 1.18]) [49]. In 2019, Aune and colleagues studied the correlation between several anthropometric factors (BMI, weight, waist circumference, waist-to-hip ratio, waist circumference, and height) and ovarian cancer risk. Mainly, they analyzed a total of 19,825 cases among 6,681,795 participants and found that a greater BMI increased the risk of developing ovarian cancer in women (the summary RR for a 5-U increment in BMI was 1.07 (95% CI: 1.03–1.11), particularly when BMI is already high in early adulthood (age 18–29 years). However, the authors did not show whether the association between BMI and ovarian cancer risk is principally related to abdominal or hip adiposity, menopausal status, or hormone therapy [50]. A recent paper in 2019 reported that changes in adiposity in young women aged 10–18 years might impact the future risk of developing ovarian cancer. In detail, the authors stated that BMI at age 18 was positively associated with ovarian cancer risk; in fact, while every 5-unit higher BMI at age 10 was related with 16% lower risk [95% confidence interval (CI) 0.74–0.96], every 5-unit higher BMI at age 18 was associated with 17% higher risk (95% CI 1.03–1.33) [51].

#### 2.5.3. Cancer of the Corpus Uteri (Endometrial Cancer)

Endometrial cancer is considered the second most common and the fourth leading cause of death related to gynecological cancers, with 417,367 new reported cases and 97,370 deaths worldwide [7]. Among gynecologic cancers, endometrial cancer is considered one of the first malignancies that is strongly associated with the obesity epidemic [52]. In 2015, a meta-analysis evaluated the association between several anthropometric factors and endometrial cancer risk. In particular, in this study, more than 22,300 cases were analyzed, and it emerged that women with a high BMI may develop endometrial cancer (RR for a 5-unit increment in BMI was 1.54 with 95% CI 1.47–1.61) and this positive association was slightly stronger in postmenopausal women [53]. Another meta-analysis including 20 prospective cohort studies and 20 case-control studies involving 32,281,242 participants confirmed that an increase in BMI was related to an augmented risk of endometrial cancer. In fact, obese and overweight women have a higher possibility of developing endometrial cancer in comparison with people with a normal weight, about 2.5 and 1.5-fold, respectively. In fact, the estimated RR and OR of endometrial cancer was 2.54 (95% CI: 2.27, 2.81) and 3.33 (95% CI: 2.87, 3.79) for obese, and 1.34 (95% CI: 1.20, 1.48) and 1.43 (95% CI: 1.30, 1.56) for overweight [54]. The link between excess body weight (EBW) and endometrial cancer risk was also reported by Zhang et al. in 2013, showing that the estimated RR for EBW was = 1.62, 95% CI, and 1.39–1.89; for obesity, it was RR = 2.54, 95% CI, 2.11–3.06; and for overweight, it was RR = 1.32, 95% CI, 1.16–1.50. Importantly, the authors also demonstrated that the positive association between endometrial cancer risk and excessive body weight was independent of several factors, such as a history of diabetes, hormone therapy, age at menarche, and age at menopause [55]. This clinical data fits well with the documented positive correlation between leptin circulating levels and endometrial cancer [55].

## 3. Main Mechanisms and Factors Involved in Obesity-Associated Cancers

**Figure 2:**
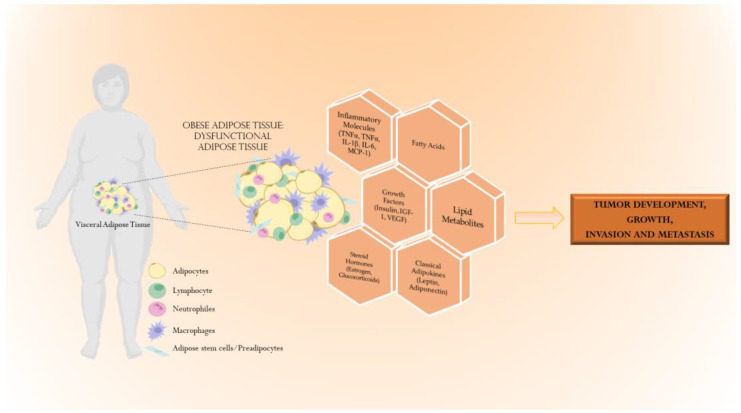
Mechanisms underlying the intricate relationship between obesity and cancers. The obese adipose tissue, characterized by hypertrophic and hyperplastic adipocytes, comprises diverse cell types such as adipocytes, adipose stem cells, endothelial cells and immune cells, and it is associated with the altered secretion of different bioactive molecules with autocrine, paracrine and endocrine functions (i.e., growth factors, adipokines, pro-inflammatory molecules, fatty acids, lipid metabolites and steroid hormones) that contribute to cancer’s development, growth, invasion and metastasis. In the figure it has been reported the visceral adipose tissue rather than the subcutaneous adipose tissue, that is known to be mainly involved in the tumorogenesis (as reviewed in Crudele et al.) [57].

Although the strong connection between obesity and cancer has been epidemiologically reported, the molecular mechanisms underlying the intricate complexity of obesity and the multifaceted abnormalities of its oncogenic drivers are still under investigation. Obesity is a multi-layered metabolic disorder characterized by excess fat storage and adipocyte mass, which induces: (i) local changes, including chronic low-grade inflammation and altered adipokine/cytokine’ secretion; and (ii) systemic disorders concerning an imbalance in insulin levels, abnormalities of the IGF-I axis, changes in hormone biosynthesis and pathway, impaired levels of adipokines, and inflammation-related mediators such as Interleukin (IL)-1β, and Tumor Necrosis Factor (TNF)-α and IL-6 (as reviewed in [56]). The local and systemic consequences of obesity-related host factors may deeply impact the survival and proliferation of cancer cells affecting the tumor cell phenotype itself and the surrounding microenvironment (Figure 2).

This still unexplored intricate connection between obesity and cancer is becoming fundamental to potentially reduce and prevent obesity development in subjects, or accurately determine patients with this disease. With these aims in mind, in the last decade, assessing anthropometric measurements, mainly height and weight, which is helpful for the calculation of BMI, was considered enough. However, BMI cannot discriminate between subcutaneous adipose tissue (SAT) and visceral adipose tissue (VAT), which each have different implications in tumor development and progression. Although SAT is the largest deposit of white adipose tissue, VAT is predominantly involved in the interaction between obesity and cancer since it is more metabolically active, produces more adipokines than SAT, and is also very close to internal organs and directly connected to the portal vein. For all these reasons, it is crucial to also assess VAT in several types of cancer, as we previously reported in the epidemiological studies. Thus, an evaluation, together with BMI, of the amount and site of deposition of adipose fat using computed tomography and magnetic resonance imaging was carried out, with a particular focus on VAT (Figure 2, reviewed in Crudele et al.) [57].

### 3.1. Inflammation

Several experimental studies have reported that adipose chronic inflammation affects tumor initiation, growth, and progression, promotes angiogenesis and metastasis, and leads to a poorer outcome and reduced therapeutic response in patients [58,59,60,61,62,63]. In fact, low-grade chronic inflammation is one of the most recognized bridges between obesity and cancer. Obesity is characterized by hyperplasia and/or hypertrophy of adipocytes, which causes the release of different inflammatory molecules, either by the tissue resident cells (adipocytes) themselves or the resident immune system [64,65,66]. Furthermore, the enormous increase in adipocyte size is accompanied by a high frequency of adipocyte death due to an insufficient supply of oxygen (hypoperfusion) in the face of widely expanding adipose tissue (AT) [67]. Furthermore, adipocyte death may promote infiltration of macrophages and, subsequently, AT inflammation [68]; hence, obesity may result in a major recruitment of macrophages [69]. Moreover, the dead adipocytes are surrounded by macrophages, recruited with the aim of engulfing the dead cells, creating crown-like structures (CLS), described for the first time by Cinti et al. in 2005, which contribute to maintain the pro-inflammatory status [69,70]. Despite this evidence, the direct or indirect contribution of macrophages to adipocyte death is still under investigation. One hypothesis assumes that adipocytes secrete several soluble factors, such as monocyte chemoattractant protein-1 (MCP-1) and leptin, which sustains macrophage accumulation and activation. On the other hand, activated macrophages release Tumor Necrosis Factor alpha (TNF-α), which induces free fatty acids (FFAs) release from adipocytes. FFAs, by binding activated toll-like receptors (TLRs) on the macrophage and adipocyte’ surfaces, promote the release of pro-inflammatory molecules, such as interleukin (IL)-1β. This paracrine loop between macrophages and adipocytes maintains the low-grade inflammatory state in obese patients [71,72]. Importantly, most of these obesity-related inflammatory molecules are associated with poor outcome in several cancers, such as breast cancer [64,69,73,74,75,76], endometrial and cervical cancer [63,77], colorectal cancer [78,79,80,81,82], and brain cancers [83,84,85,86,87,88,89,90].

Moreover, the inflammasome activation in AT has been described as one of the mediators of inflammation in obese patients [91]. Inflammasomes are multiprotein complexes that simulate the secretion of IL-1β and IL-18 via Caspase 1 (Casp-1)-mediated cleavage in response to pathogen-associated molecular patterns (PAMPs) and danger-associated molecular patterns (DAMPs). Inflammasomes consist of Nod (Nucleotide-binding and oligomerization domain)-like receptors (NLRP1, NLRP3, and NLRC4), the adaptor apoptosis-associated speck-like protein containing a caspase-recruitment domain (ASC), and the enzyme caspase-1 (CASP-1) [92]. A study conducted by Yin et al. demonstrated that obese subjects expressed high levels of inflammasome-related genes, such as NLRP3, CASP1, IL1B, CCL2 (C-C Motif Chemokine Ligand 2), and TNF, compared to lean subjects [93]. In addition, the role of inflammasome in cancer has been described [64,94,95,96,97]; however, so far, there is no evidence for the role of inflammasome activation in obesity-associated cancers, although inflammasome activation may lead to insulin resistance, one of the most independent risk factors for several types of cancer [98,99,100]. Furthermore, IL-1β is under investigation as a key molecule in inflammasome activation in obesity-associated cancers [64,101]. To date, a more comprehensive and accurate study of inflammasome could be a novel avenue to harness the treatment of obese patients.

### 3.2. Insulin/IGF-1 Axis

Obesity, especially determined by an excess of fat in the abdomen and around the visceral organs, known as visceral fat, has a key role in insulin resistance syndrome, characterized by hyperinsulinemia, insulin resistance, and/or hyperglycemia. A growing body of evidence correlates chronic hyperinsulinemia with various types of cancers, such as colorectal cancer [102,103], pancreatic cancer [104,105], endometrial cancer [106], and breast cancer [107]. In fact, researchers have demonstrated that insulin promotes carcinogenesis through a direct effect on insulin receptors or indirectly by affecting the levels of other modulators, such as the insulin-like growth factor (IGF) family of receptors, sex hormones, and adipokines [108,109]. The IGF system includes IGF-I and IGF-II, the type I and type II receptors (respectively, IGF-IR and IGF-IIR), and a family of IGF binding proteins (IGFBPs) that specifically bind IGFs [110]. Noteworthy, IGF-I, one of the most important growth factors released by adipocytes, sustains their differentiation and metabolic regulation [111]. On the other hand, altered levels of this molecule may stimulate tumor malignancies. Particularly, IGF-1 binds to IGF-1R, a tyrosine kinase receptor, which activates its downstream signal effectors, comprising Ras/Raf/ERK and PI3K/Akt/mTOR, known to be involved in cell growth, proliferation, and in various types of cancers [112,113]. In fact, previous studies suggest that higher levels of IGF-1 are positively associated with the risk of breast cancer [114,115,116,117,118], prostate cancer [119,120,121], and colorectal cancer [122]. In aggregate, these data emphasize the important role of the insulin/IGF-1 axis in tumor biology.

### 3.3. Adipokines

A key role in the physiopathology of obesity-related cancer is represented by a wide variety of heterogeneous bioactive molecules. Among them, the adipokines, a large group of heterogeneous peptides mainly produced by adipose tissue, are an emerging landscape in the link between obesity and cancer. To date, more than 100 different adipokines have been discovered, of which adiponectin and leptin are the most studied to be implicated in obesity-related tumorigenesis and progression. Adiponectin is the most abundant adipokine in plasma and its secretion is strongly related to the circulating levels of other hormones. Interestingly, an inverse association between adiponectin and fasting plasma insulin has been demonstrated [123]. In fact, low levels of adiponectin have been associated with obesity and insulin resistance. This is supported by several reports showing that anti-diabetic drugs, including peroxisome proliferator-activated receptor gamma (PPAR-γ) agonists belonging to the thiazolidinedione’s class and metformin, can affect serum adiponectin concentrations, enhancing its expression and secretion [124,125,126,127]. Hence, recently, researchers have demonstrated that adiponectin plays a pivotal role in the development and possible progression of several types of obesity-associated cancers, such as breast [43,128,129,130,131,132,133,134,135,136,137,138], endometrial and ovarian [139,140], and colorectal [141,142]. Meanwhile, several pieces of evidence have shown that leptin, whose circulating levels proportionally increase with fat mass, directly or indirectly impacts the biology of several cancers. Here, we reported the clinical, in vitro, and in vivo studies demonstrating the strong relationship between adipokine and cancer, as summarized in Table 1.

## 4. In Vitro, In Vivo and Clinical Studies Relating Leptin and Obesity-Associated Cancers

### 4.1. Leptin Biological Activities

**Figure 3:**
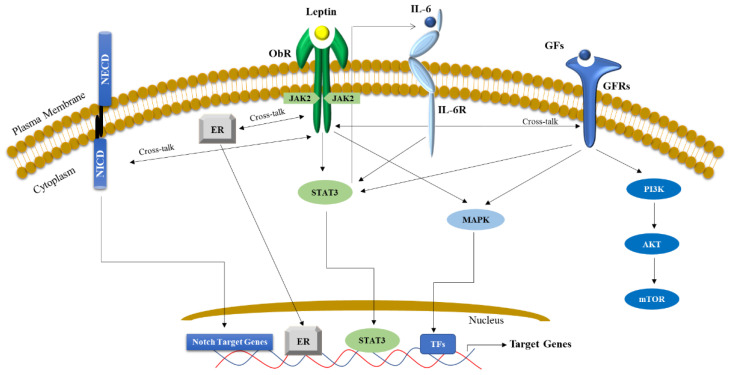
Molecular mechanisms through which leptin impacts cancer biology. Leptin and its own receptor (ObR) are expressed in different cancer cells and mainly lead to the activation of the JAK/STAT pathway, a potent signaling cascade that controls various biological processes (i.e., cell proliferation, migration and invasion). Leptin/Leptin Receptor can crosstalk with: (i) Notch by inducing the cleavage of the intracellular domain of the receptor that regulates the transcription of several genes mainly involved in stemness; (ii) Estrogen receptor (ER) alpha sustaining its activation and signaling, (iii) Interleukin (IL)-6 that binds its receptor (IL-6R) and potentiates the STAT signaling and (iv) several growth factor receptors (GFRs) such as VEGF, IGF and EGF Receptors. Mostly, the interaction with these multiple oncogenic signaling also contributes to the activation of various signal transduction pathways, such as PI3K/Akt/mTOR and MAPK, and further supports STAT3 activation, fundamentals to sustain tumor growth and progression. NECD: Notch extracellular domain; NICD: Notch Intracellular domain; TFs: Transcriptional Factors; GFs (Growth Factors).

Leptin is a 16-kDa pleiotropic peptide hormone encoded by the obese (Ob) gene, whose secretion increases proportionally to fat cell mass. Normally, leptin regulates food intake and appetite, energy expenditure and energy homeostasis, immune response, and reproductive processes. Leptin exerts its action by binding its own receptor (ObR), a member of the class 1 cytokine receptor family, which is implicated in several processes linked to cancer progression, including cell proliferation, metastasis, angiogenesis, and chemoresistance [169,208,209,210,211,212,213]. The downstream effects of leptin signaling, through the activation of specific signaling pathways in tumor cells, such as the JAK/STAT3 and PI3K/AKT pathways, can promote carcinogenesis [83,182,214,215,216,217]. Moreover, the crosstalk between leptin with NOTCH, IL-6 receptor, and other different growth factor receptors (VEGFR, IGFR and EGFR) has been well documented to promote the development and progression of several cancer cells (Figure 3) [200,218,219].

#### Colorectal Cancer

- In vitro and in vivo studies

In vitro studies unraveled the key role of leptin in CRC by demonstrating how leptin is able to induce migration and invasion in CRC cell lines [220,221]. Interestingly, several studies reported that leptin is able to induce proliferation and invasiveness through the activation of MAPKs, PI3K, NF-κB, and STAT3 signaling in CRC cell lines [143,144,222]. Leptin could also increase proliferation and inhibit apoptosis through the PI3K/Akt/mTOR pathway in HCT-116, a widely used experimental model for colon cancer. Therefore, the leptin signaling pathway, through JAK/STAT, has been found activated in colorectal adenoma, along with transcriptional regulation of STAT3 downstream target molecules [144]. Interestingly, it has been demonstrated that leptin might induce the secretion of IL-6 and TNF-α as well as promote proliferation in colon epithelial cells, in a mTOR-dependent way [81]. In addition, it has been reported that the use of leptin-derived peptides has been shown to reduce tumor growth in CRC mouse models [153,154].

- Clinical studies

Several investigators have demonstrated that leptin circulating levels were significantly higher in colorectal cancer patients [13,14,15]. Moreover, Erkasap et al. demonstrated an increased ObR expression in metastatic colorectal tissues compared to local colorectal cancer tissues [150]. Furthermore, it has been revealed that leptin increased expression leads to higher metastatic potential and promotes neoangiogenesis [145,146]. On the other hand, additional investigators found lower leptin levels in CRC patients [147,148], whereas Tessitore et al., in a prospective study, observed in CRC patients that leptin levels were similar to those of the control [149].

### 4.2. Lung Cancer

- In vitro and in vivo studies

Despite the debated role of leptin in lung cancer, in vitro and in vivo evidence has shown that this adipokine could promote the growth, migration, and invasion of lung cells. In fact, leptin might affect the epithelial-to-mesenchymal transition of A549, a widely used model for the lung cancer cell line [25,155,159]. Mainly, it has been proven that leptin, through the activation of the MAPK signaling pathway, induced the expression of genes encoding mesenchymal phenotype markers such as Vimentin and Zeb-1, while reducing the levels of cadherin and keratin, which are well-known epithelial markers. Thus, leptin was able to promote the migration and invasion of lung cancer cells [155]. In addition, leptin could sustain lung cancer cell proliferation by the activation of the PI3K/AKT/mTOR signaling pathway and through the down-regulation of the p53 signaling pathway [25]. In line with this evidence, it has also been proved that leptin deletion results in reduced cell growth and induces apoptosis in NSCLC cell lines, partially involving the Notch and JAK/STAT3 signaling pathways [156]. Furthermore, Jiang and coworkers also highlighted the potential role of leptin in promoting brain metastasis in lung adenocarcinoma through a mechanism involving the long coding RNA lnc-REG3G-3-1 and the miRNA-215-3p [159]. Moreover, leptin secreted from cancer-associated fibroblasts isolated from NSCLC patients acting in a paracrine way can sustain NSCLC cell proliferation and migration by activating both the PI3K-AKT and MAPK-ERK signaling axes [157]. The carcinogenic role of leptin in the lung tumor microenvironment was also evidenced by Wang and colleagues. The authors showed that leptin secreted from bone marrow-derived stem cells through the activation of IGF1 signaling pathway induces resistance to erlotinib, an inhibitor of EGFR introduced into the first-line treatment for NSCLC patients [158].

- Clinical studies

Leptin has been widely recognized as an important player in cancer biology in other malignancies, while its action in lung cancer is still controversial and under investigation. Noteworthy, it has been shown that the expression of both leptin and leptin receptor was higher in NSCLC compared to the normal counterpart tissues [22,23,24,25]. Particularly, Karatas et al., in 2017, demonstrated that increased serum leptin levels were mainly correlated with NSCLC patients affected by adenocarcinoma [223]. In contrast, Ann and colleagues found that leptin serum levels were lower in lung cancer patients compared to normal subjects. Moreover, they did not report any significant differences in leptin values related to clinicopathological parameters, sustaining that leptin has no prognostic value in lung cancer patients [160].

### 4.3. Glioma Cancer

- In vitro and in vivo studies

Ferla et al. demonstrated that two different human GBM cell lines, LN18 and LN229, express ObR and are able to secrete leptin, which acts as a pro-angiogenic stimulus for endothelial cells. In line with these findings, a peptide ObR antagonist can block proangiogenic and growth induced-leptin effects on endothelial cells. Interestingly, the same authors showed significantly reduced tumor cell proliferation by using the ObR antagonist in combination with a VEGF pathway-targeting drug [161]. Furthermore, Han et al. demonstrated, in in vitro and in vivo studies, that the cells positive to leptin receptor (ObR+) exhibited temozolomide (TMZ) resistance characteristics. Likewise, the TMZ-resistant cells (U87R) displayed a high expression of ObR [162]. Moreover, they demonstrated that TMZ resistance in ObR+ cells were correlated with stem/progenitor cell properties, in which the STAT3-mediated sex-determining region Y-box 2 (SOX2)/octamer-binding transcription factor 4 (OCT4) signaling axis plays a key role [61,162]. Furthermore, it has been demonstrated that LDFI, a peptide able to block ObR, along with AG490, a JAK2/STAT3 inhibitor, is able to reduce leptin-induced NOTCH1 overexpression and transcriptional activity in U87 and T98 glioma cell lines. Thus, the drugs reduced neurosphere forming efficiency, self-renewal frequencies, and clonogenic ability [83]. Moreover, leptin induced an increase in migrated and invaded cells in C6 rat GBM cells through the cascade of p38 mitogen-activated protein kinase (MAPK) and Nuclear Factor-kB [164].

- Clinical studies

Different studies have demonstrated how leptin and its receptor are expressed in GBM human primary tissue according to the degree of malignancy [83,162,163,165]. Recently, a positive correlation between circulating levels of leptin and glioma has been found [34]. Furthermore, it has also been evidenced that GBM cells consisting of vasculogenic mimicry (VM), the vascular channels lacking endothelial cells that are characterized by tumor cells with cancer stem cell features, were positive correlated with leptin expression and its receptor. Interestingly, ObR positive GBM specimens with VM were associated with tumor metastasis and a lower overall survival rate [166].

### 4.4. Postmenopausal Breast Cancer

- In vitro and in vivo studies

In vitro findings have shown the pro-oncogenic impact of leptin, including cell proliferation, transformation, apoptosis regulation, self-renewal, and reduced sensitivity to breast cancer treatments (reviewed in [224,225,226]). Particularly, leptin may act by (i) binding its own receptor (ObR) or (ii) by interacting with other different mediators known to be involved in breast cancer biology (e.g., ERα, growth factors, Notch, and inflammatory cytokines) [40]. Moreover, it has been demonstrated that leptin may specifically amplify estrogen signaling in two different ways, (i) by increasing aromatase gene expression or (ii) by directly transactivating ERα [167,168]. In addition, it has been shown how leptin signaling may potentiate ERα’s role in a lysine to arginine mutation at residue 303 (K303R) within the hinge domain of ERα, which promotes the growth and progression of breast cancer, especially in K303R-associated breast cancer [169]. Interestingly, it has been demonstrated in a model of normal mammary epithelial cells (MCF10A) that leptin may induce epithelial-to-mesenchymal transition, an essential process for metastasis development. Moreover, the same authors demonstrated in normal breast tissues a positive correlation between ObR and mesenchymal markers (such as vimentin, N-cadherin, matrix metalloproteases, and transcription factors such as Snail, Slug, Twist, and Zeb), and a negative correlation between ObR and epithelial markers (such as E-cadherin, occludins, and claudins). These findings suggest that leptin, by altering the normal mammary gland epithelium, may induce the development of breast tumors [172]. It has been largely demonstrated that a small population of cancer cells, referred to as breast cancer stem cell (BCSC), drive and sustain tumor growth, tumor relapse, and resistance to therapy [227,228,229]. In line with these observations, other findings demonstrated the pivotal role of Ob/ObR signaling in the breast cancer stem cell (BCSC) phenotype. Indeed, the use of an antagonist of the leptin receptor, the LDFI, completely reversed the BCSC phenotype sustained by leptin [171]. In the same vein, a specific nanoparticle-linked antagonist was able to reverse leptin-induced effects in terms of proliferation, stem cell properties, and response to chemo drugs [170]. Finally, according to these data, Thiagarajan et al. showed that ObR/STAT activation is essential for maintaining CSC metastatic properties in breast cancer cells [230]. Finally, leptin has been observed to influence cell cycle. Notably, leptin acts by reducing the number of cells of the G0/G1 phase and incrementing the number of breast cancer cells with the S and G2/M phases, thus supporting cell division and proliferation. The effect of leptin on tumor progression relies on the increased expression of antiapoptotic proteins, such as Bcl-2 and Bcl-xl, which suppress the programmed cell death of breast cancer cells [173]. Recently, in mammary tissues of a rat model of breast cancer driven by diet-induced obesity, increased expression of the leptin/leptin receptor (Ob/ObR) along with signaling activation were found. In addition, an increase in tumor aggressiveness was observed in the same mice [176]. In SCID (severe combined immunodeficiency)/beige mice implanted either with ER-positive MCF-7 breast cancer cells: (i) alone, (ii) mixed with obese adipose stromal/stem cells (obASCs) isolated from obese patients, or (iii) mixed with obASCs silenced for leptin gene (leptin shRNA obASCs), a reduction in tumor growth and a decreased number of lung and liver metastases were found compared to those not silenced for the leptin gene [177]. Cleary et al. demonstrated that two in vivo models of obesity, the mouse mammary tumor virus (MMTV)-TGF-α/Lep(ob)/(ob) (leptin-deficient) and MMTV-TGF-α/ObR(db)/(db) (leptin receptor-deficient) mice, did not develop mammary tumors compared to wild-type mice [178,179]. Moreover, MMTV-Wnt-1 leptin-deficient mice (Lepob/ob) showed a reduction in tumor growth compared to wild-type mice [180]. More recently, a correlation between hyperleptinemia and responsiveness to endocrine therapy in breast cancer has been found. In fact, in a model of obese mice with hyperleptinemia, increased tumor progression and a poor response to tamoxifen were found compared to non-obese mice [181]. Recently, it has also been found that the integrity of Ob/ObR signaling is necessary not only to sustain breast cancer tumor growth but also for the interaction between cancer cells and the surrounding tumor microenvironment. Particularly, it has been found that the absence of the leptin receptor reduced the recruitment of macrophages and inhibited breast cancer growth and progression [182].

Besides its role in tumor cell growth, leptin has also been recognized as one of the most important mediators in cell-to-cell communication within the breast tumor microenvironment, mainly in the interaction between stromal cells (i.e., adipocytes, cancer-associated fibroblasts, endothelial cells, and macrophages) and neoplastic cells (as reviewed in [225]). Recently, in the context of the breast tumor microenvironment, Giordano et al. demonstrated in 2019 that leptin modulates the biogenesis of extracellular-vesicles, phospho bilayered vesicles secreted by all cell types that play an important role in cellular communication. Leptin, through Heat Shock Protein 90, is able to modulate the expression, at the post-transcriptional level, of the tumor susceptibility gene 101, known to be a key component of the endosomal sorting complex required for transport I [174]. Additionally, the same group reported that leptin affects the extracellular vesicle cargo, sustaining the expression of proteins mainly related to mitochondrial machinery and activity, thus modifying the behavior of recipient breast cancer cells and macrophages in terms of metabolism and energy production [175].

- Clinical studies

The overexpression of leptin and its own receptor (ObR) has been found in high-grade breast cancer and is positively correlated with poor prognosis and distant metastasis [39,40,41,42,43,44]. Moreover, Kaplan–Meier survival analysis correlated leptin receptor expression with reduced overall survival in breast carcinoma patients, especially in basal-like cancer subtypes [171].

### 4.5. Gynecological Cancer

#### 4.5.1. Cervical Carcinoma

- In vitro and in vivo studies

In the last few years, cervical carcinoma CSCs have been raised as a potential target for this neoplasia. In this context, it has been shown that leptin could also be involved in the maintenance of the CSC phenotype [197]. In fact, it has been reported that using an inhibitor of ERK1/2, a well-known downstream signaling pathway of leptin, could drastically attenuate the stem cell properties mediated by TGF-β1/CK17 [231]. In aggregate, these data may suggest the importance of highlighting the actions of CSC in progressive and metastatic cervix cancer. Moreover, further studies are necessary to better elucidate the role of leptin in cervical carcinoma.

- Clinical studies

High ObR expression is correlated with poor outcomes in women with cervical cancer, whereas high leptin levels are associated with favorable outcomes [197,198]. Lebrecht et al., by examining VEGF and leptin serum levels in 84 women with cervix carcinoma, 28 patients with cervical intraepithelial neoplasia (I–III), and 35 healthy women, found a positive correlation between VEGF and cancer and tumor stage. However, they did not notice any significant difference in serum leptin levels among the different groups [199]. Subsequently, Yuan et al. demonstrated that leptin levels were increased in cervix carcinoma cells in a dose-dependent fashion and this correlated with increased protein and mRNA levels of c-myc, which is a well-known oncogene. Moreover, the same authors performed a study assessing leptin levels in 80 patients with cervix carcinoma. Interestingly, they found that leptin was positively correlated with a higher grade of cervical carcinoma along with an increased expression of c-myc and bcl-2 antiapoptotic genes. Finally, they demonstrated that silencing leptin decreased leptin-induced proliferation, along with reduced expression of c-myc and bcl-2. Thus, the authors demonstrated that leptin plays a role in cervical carcinoma growth and progression [48].

#### 4.5.2. Ovarian Cancer

- In vitro and in vivo studies

A growing amount of evidence has confirmed that leptin treatment significantly induces cell growth and proliferation in several ovarian cancer cell lines, including SKOV3, OV-90, A2780, BG-1, and OVCAR3 cells [185,186,187,188]. Particularly in the ovarian cancer cell line OVCAR-3, Chen et al. demonstrated that leptin was involved in the regulation of cell proliferation through the activation of PI3K/AKT pathways along with the mitogen-activated protein kinase (MEK)/ERK1/2 signaling pathways. Interestingly, the same authors observed leptin-induced growth in a time- and dose-dependent manner concurrent with an increase in STAT3 and AKT phosphorylation [185]. It has been demonstrated that leptin may stimulate cell migration by activating the matrix metalloproteinase MMP-9 [191]. In fact, another investigation suggested that silencing MMP-7 in SKOV3 cells promoted a reduction in the leptin-induced activation of MMP-9 [193]. Along with MMPs, Urokinase plasminogen activator (uPA) has been studied to participate in tumor cell migration by degrading extracellular matrix (ECM) protein [232]. In the latter concern, Ghasemi et al. reported in different ovarian cancer cell lines (OVCAR-3, SKOV3, and CaoV-3) a leptin-induced invasion mediated via uPA upregulation [190]. Moreover, two different studies demonstrated the involvement of RhoA/ROCK (cytoskeletal regulators), PI3K/AKT, JAK/STAT pathways, and nuclear factor kappa-B (NF-κB) activation in leptin-induced migration and invasion [192,193]. Furthermore, the same authors demonstrated how leptin may be involved in the maintenance of stemness and may display a mesenchymal phenotype in ovarian cancer cells, thus contributing to tumor progression [192]. Recently, Fiedor et al. demonstrated that ovarian cancer cells expressed a higher level of ObR (almost 50%) compared to their normal counterparts. Moreover, by using specific leptin receptor antagonists, they found that these compounds were able to inhibit the leptin-induced proliferation of ovarian cancer cells [189]. Mouse ovarian cancer ID-8 cells implanted in female C57B6 mice fed a high energy diet (HED, 60 kcal% fat—fed ad libitum), a regular diet (RD, 7.2 kcal% fat—fed ad libitum), and a caloric restriction diet (CRD, 30% reduced from normal intake) displayed a more extensive tumor volume at all the peritoneum-related organs and metastatic sites in the HED group. Interestingly, it was accompanied by an increase in the circulating levels of leptin, along with several inflammatory cytokines and hormonal molecules (IL-6, MCP-1, VEGF, and IGF-1. Conversely, the CRD group showed the reverse profile [233]. In another in vivo study, it was observed that C57B6 mice fed with HED and CRD and treated daily with the anti-diabetic agent metformin displayed a significant reduction in tumor volume along with a reduction in circulating levels of IGF-1, IL-6, and leptin in both plasma and ascetic fluid, similar to the CRD mice [194]. Overall, in vivo investigations demonstrated the positive correlation between obesity and ovarian cancer [234,235], although some of those did not investigate circulating leptin levels.

- Clinical studies

The role of leptin is still controversial in ovarian cancer. Indeed, one study demonstrated that overweight ovarian cancer patients who exhibit higher levels of leptin as well as ObR expression in serum and ascites isolated from metastatic patients demonstrate worse overall survival (OS) [192]. On the other hand, a clinical trial showed that the ovarian cancer group exhibited lower levels of leptin compared to the group with benign ovarian masses in pulled premenopausal and postmenopausal women [195]. Accordingly, a Kaplan–Meier analysis suggested that leptin overexpression is a positive prognosticator of disease-free survival (DFS) along with disease-specific survival (DSS) in ovarian cancer patients [196].

#### 4.5.3. Cancer of Corpus Uteri (Endometrial Cancer)

- In vitro and in vivo studies

Leptin treatment induced cancer cell proliferation in six different endometrial cancer cell lines, by which: (i) Ishikawa, (ii) ECC-1 cell lines, both derived from well-differentiated endometrial adenocarcinoma, (iii) HEC-1A, (iv) substrain HEC-1B derived from moderately differentiated endometrial adenocarcinoma, (v) RL95-2 derived from moderately differentiated adenosquamous carcinoma of the endometrium, and (vi) AN3CA derived from undifferentiated endometrial adenocarcinoma [201]. Moreover, it has been shown that in Ishikawa cells, leptin is able to induce cancer cell growth in a time- and dose-dependent manner. Interestingly, the same authors showed an increased cancer cell invasion after treatment with leptin at a dose of 100 ng/mL [203]. In addition, leptin exposure (100 ng/mL) was demonstrated to induce endometrial cancer cell proliferation and migration mostly in two distinct types of EC: Type 1 (or endometrioid adenocarcinomas), characterized by excess estrogen in the body (around 3–3.5 fold change), and Type 2, which are not linked to excess estrogen (2–2.5 fold change) [200]. Furthermore, the crucial role of leptin in the cell cycle through the increased S-phase cell population, along with an increase in cyclin D1 (a critical modulator of G1/S transition) and a decrease in p21, known as cyclin-dependent kinase inhibitor 1, upon leptin exposure, has also been demonstrated [202]. Consequently, it has been reported that the higher rate of S-phase progression, along with cancer cell proliferation and migration, was evidenced to a greater extent in Type 2 endometrial cancer treated with leptin compared to type 1 [200]. In addition, it has been demonstrated that leptin protects Ishikawa and HEC-1A cells from apoptosis [204].

- Clinical studies

A positive correlation between leptin-circulating levels and the risk of endometrial cancer has been documented [205,206]. Interestingly, some investigators found in endometrial cancer tissue higher circulating levels of leptin and its receptor (Ob/ObR) in comparison with normal endometrial tissue [55]. Furthermore, Cymbaluk-Płoska et al. showed that a higher leptin level was associated with lymph vessel involvement and poor neoplastic differentiation [207].

### 4.6. Agonists and Antagonists of Leptin/Leptin Receptor for Future Therapeutic Approaches

**Figure 4:**
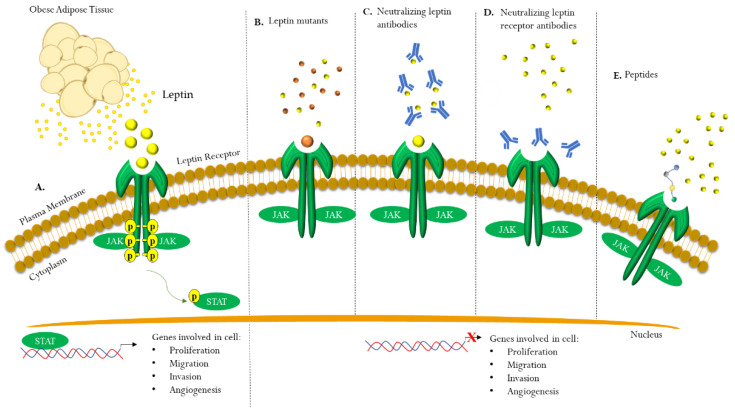
Schematic representation of the current strategies to impair leptin/leptin receptor signaling. In obese patients, hypertrophic and hyperplastic adipose tissue expansion leads to an increased local and systemic production of the adipokine leptin. Leptin, through the binding to its own receptor (ObR) expressed on cancer cell surface activates the canonical JAK2/STAT3 signal transduction pathway, with consequently induction of transcriptional machinery of several key genes determinant for tumor progression (**A**). Leptin mutants (**B**), neutralizing antibodies against leptin (**C**) or leptin receptor (**D**), leptin receptor antagonists (mainly small peptide) have been developed to block leptin/leptin receptor signaling (**E**).

Besides its important physiological role, leptin is also involved in tumor development and progression. Thus, it is becoming crucial to design novel therapies that may impair leptin signaling in malignancies. In the last few decades, several leptin-related agonists and antagonists have been developed, and among them, leptin mutants, leptin receptor antagonists, and neutralizing antibodies have been shown to be promising (Figure 4).

- Leptin mutants

The first therapeutic approach to block leptin signaling was reported in 1997 by Verploegen et al., who demonstrated how the substitution of arginine with glutamine at position 128 of human leptin completely inhibits leptin/leptin receptor binding and blocks its biological activity, showing a promising anti-cancer effect [236]. Mostly, leptin antagonists have been developed by replacing the aminoacids of the sequence 39–42 with alanine residues as the triple mutein L39A/D40A/F41A (LDF or Lan-1) and the quadruple mutein L39A/D40A/F41A/I42A (LDFI or Lan-2) [237]. In 2017, Fiedor and collaborators identified super-active human leptin antagonists (SHLA, D23L/L39A/D40A/F41A mutant), triple Lan1, and quadruple Lan2 leptin mutein as promising treatments for ovarian cancer, particularly the folliculoma type. They reported the successes of all ObR antagonists used in reversing the leptin-stimulatory effects on the proliferation of several cell lines and impairing the crosstalk between leptin and estrogen signaling [189].

- ObR antagonists

Noteworthy, the small peptide based on the wild-type sequence of the leptin binding site I (LDFI) synthetized by Andò and colleagues in 2015, by competing with leptin, blocks leptin/leptin receptor signaling and has shown important results in vitro and in vivo in several types of cancer, including breast, seminoma, and glioblastoma [83,171,174,211,238]. The inhibition of the leptin-mediated signaling pathway correlates with decreased breast cancer cell proliferation and stem cell activity, as well as tumor growth reduction, as reported in the xenograft experiments [171,238]. Moreover, the LDFI antagonist hampers leptin’s role in cell-cell paracrine interactions by reducing exosome release from breast cancer cells [174]. The inhibitory effects of LDFI on leptin signaling were also observed in seminoma [211] and, more recently, in human glioblastoma cells, U87 MG [83]. Another ObR antagonist peptide named Aca1 has been shown to reverse the mitogenic and angiogenic effects induced by two different glioma cell lines (LN18 and LN229) on endothelial cells [161]. In 2017, Harmon et al. developed a leptin peptide receptor antagonist LPrA2, a potent inhibitor of leptin signaling conjugated with iron oxide nanoparticles (IONPs) as a nanoparticle delivery system. They found that IONP-LPrA2 2 reduced leptin-induced cell proliferation in human breast cancer cells through the decreased expression of pSTAT3 and Cyclin D1. Moreover, this innovative nanoparticle delivery system reduced the leptin-induced stem cell phenotype and, in combination with chemotherapeutic drugs, affected the cell cycle by blocking DNA synthesis in the S phase. Thus, it has been suggested that the synergistic effects between IONP-LPrA2 and chemotherapeutics may be a novel potential combined treatment for breast cancer patients, particularly for triple-negative subsets [170]. Later, the same approach was used by Daley-Brown and colleagues. They found that IONP-LPrA2 re-sensitizes endometrial cancer cells to the chemo-drug Paclitaxel [200].

- Neutralizing antibodies

Although all peptides showed important inhibitory effects on different types of tumors, specific anti-leptin-receptor monoclonal antibodies (anti-LR mAbs) could be more efficient due to their high molecular mass and longer half-life in the circulation. They also have a greater affinity for the ObR receptor. Recently, Munikumar and collaborators designed novel peptidomimetics with in silico studies derived from an existing 9F8 Fab monoclonal in order to improve its efficacy. They proposed six novel lead peptides with greater potential compared to native 9F8 Fab peptides in terms of binding affinity in docking and molecular dynamics simulations, but these still need to be tested in vitro and in vivo [239]. Furthermore, the administration of combined polyclonal and monoclonal anti-leptin receptor antibodies has been demonstrated to be an antagonist in the presence of leptin, preventing its binding to the receptor [240]. In 2018, Zabeau et al. identified a camelid single-domain antibody able to interfere with the cross-talk between the leptin receptor (LR) and the EGFR. Importantly, this molecule affects the immunomodulatory activity of leptin but not its canonical metabolic functions [241]. The same group designed three classes of neutralizing nanobodies and 15-kDa monomeric antibodies with a single antigen binding domain, targeting the extracellular part of the mouse ObR. Mainly, the nanobodies, by blocking different subdomains, inhibited ObR activity. In fact, the molecules against CRH2 prevented leptin binding, while the compounds against the Ig-like and FNIII (fibronectin type III) domains affected receptor activation, but not leptin binding [242]. The development of nanobodies should overcome the limitations of antibodies due to their smaller size, allowing them to facilitate tissue penetration, and should be preferred to peptides since they mostly exhibit rapid clearance from the body through enzymatic cleavage and renal filtration.

## 5. Conclusions

Obesity, characterized by abnormal fat accumulation and the hypertrophic or hyperplastic state of adipocytes, is a severe and multi-layered public health issue involved in metabolic and cardiovascular disease as well as cancer. Several studies have concluded that obesity may amplify the risk and mortality of different types of malignancies, including cancers of the digestive tract (colorectal), post-menopausal breast cancers, and gynecological cancers (corpus uteri and ovarian). Emerging evidence also concerns other neoplasia, such as cancer of the central nervous system, gliomas, and lung cancer.

Adipose tissue, along with its chronic inflammatory status, is the major driver sustaining the association between obesity and cancer. However, some mechanisms underlying the obesity-cancer crosstalk are still under investigation. Epidemiological and clinical data recognize the adipocyte secreted hormone leptin as one of the most important mediators of the link between obesity and cancer. Indeed, leptin and the leptin receptor are usually overexpressed in cancer patients, wherein this adipokine stimulates proliferation, migratory and invasive potential, and stemness capabilities in the multiple types of tumors here discussed. Thus, a deeper knowledge of the mechanisms linking obesity to malignancies could lead to the identification of novel biomarkers as well as therapeutic interventions that optimize the clinical management of obese patients with cancer that, according to the previously mentioned pharmacological tools, require a tailored treatment strategy.

## 6. Review Criteria

This review is a collection of different studies and an analysis of information obtained on the relationship between obesity and cancer and obesity and leptin in the last few decades. The literature search was conducted using the PubMed electronic database by searching the following keywords: leptin or obesity AND cancer, leptin or obesity AND colorectal cancer, leptin or obesity AND glioma, leptin or obesity AND lung cancers, leptin or obesity AND breast cancer, leptin or obesity AND colorectal cancer, obesity AND cervical cancer, obesity AND ovarian cancer, leptin or obesity AND cancer of the corpus uteri, leptin or obesity AND endometrial cancer. We included original research articles, meta-analyses, and reviews that met our search terms, published in the last few decades and written in English. Both full articles and abstracts were taken into consideration.

## Figures and Tables

**Table 1:** Studies showing the leptin-mediated effects in obesity-associated cancers.

Type of Cancers	In Vitro and In Vivo Studies	Clinical Studies	Therapeutic Approaches
***Colorectal (CRC)***	(i) Leptin induced proliferation and invasiveness through activation of MAPKs, PI3K, NF-κB, and STAT3 signaling in HT-29 cells [81,143]. (ii) Leptin inhibited the apoptosis of HCT-116 colon cells through the PI3K/Akt/mTOR pathway [144]. (iii) Leptin promoted proliferation in a m-TOR-dependent way in IEC-6 cells [81].	(i) Leptin positively correlated with proliferation index of cancer cells and angiogenesis in CRC [145,146]. (ii) High, decreased or equal circulating leptin levels have been found in CRC patients compared to normal subjects [14,15,64,147,148,149]. (iii) Metastatic colorectal tissues showed increased ObR expression compared to local colorectal cancer tissues [150]. (iv) Leptin levels positively correlated with the risk of CRC in men but not in women [151,152].	Leptin-derived peptides (e.g., the Lp31 or LP16 peptides) have been shown to reduce tumor growth in CRC mouse models [153,154].
***Lung***	(i) Leptin promoted migration and invasion through the activation of MAPK signaling pathway, inducing the expression of mesenchymal genes (i.e., Vimentin and Zeb-1) while reducing the levels of cadherin and keratin, well-known epithelial markers [155]. (ii) Leptin induced lung cancer cell proliferation by the activation of the PI3K/AKT/mTOR signaling pathway and through down-regulation of the P53 signaling pathway [25]. (iii) Leptin deletion induced a reduced cell growth and apoptosis in non-small-cell lung cancer (NSCLC) cell lines partially involving the Notch and JAK/STAT3 signaling pathways [156]. (iv) Leptin secreted from cancer-associated fibroblasts isolated from NSCLC patients acting in a paracrine way sustained the NSCLC cell proliferation and migration by activating both the PI3K-AKT and MAPK-ERK signaling axes [157]. (v) Leptin secreted from bone marrow-derived stem cells through the activation of IGF1 signaling pathway induced resistance to erlotinib, an inhibitor of epidermal growth factor (EGF) receptor introduced into the first-line treatment for non-small-cell lung cancer (NSCLC) patients [158]. (vi) Leptin sustained brain metastasis in lung adenocarcinoma, through a mechanism involving the long coding RNA lnc-REG3G-3-1 and the miR-215-3p [159].	(i) Leptin and leptin receptor have been found overexpressed in non-small-cell lung cancer (NSCLC) compared to the normal counterpart tissues [22,23,24,25]. (ii) Leptin serum levels have been found lower in lung cancer patients compared to normal subjects [160].	N/A
***Glioma***	(i) Leptin/leptin receptor acted as pro-angiogenic stimulation for endothelial cells in GBM cells (LN18 and LN229) [161]. (ii) ObR-positive cells exhibited temozolomide (TMZ)-resistant phenotype. Likewise, the TMZ-resistant cells (U87R) displayed high expression of ObR [162]. (iii) Leptin significantly promoted U87 tumor cells growth in a time-and-dose-dependent manner [61]. (iv) Leptin increased cell DNA synthesis and promoted G(0)/G(1) phase-to-S phase transition [61]. (v) Leptin increased invasive ability in U87 glioma stem-like cells (GSCs) [83,163]. (vi) Leptin supported cell migration and invasion in C6 rat cells, through the cascade of p38 mitogen-activated protein kinase (MAPK) and Nuclear Factor-kB (NF-kB) [164]. (vii) ObR-positive cells exhibited higher tumorogenicity in vivo compared to ObR-negative cells [162].	(i) Leptin and its receptor have been found expressed in glioblastoma (GBM) human primary tissue, accordingly with the degree of malignancy [83,162,163,165]. (ii) Leptin positively correlated with glioma [34]. (iii) ObR positive glioblastoma specimens have been found associated with tumor metastasis and lower overall survival rate [166].	(i) ObR-derived peptide blocked proangiogenic and growth induced-leptin effects on endothelial cells in LN18 and LN229 cells [161]. (ii) ObR derived peptide, in combination with AG490, a JAK2/STAT3 inhibitor, reduced the NOTCH1 overexpression and transcriptional activity induced by leptin in U87 and T98 cells along with their clonogenic ability [83].
***Post-menopausal breast***	(i) Leptin amplified specifically estrogen signaling in MCF-7 cells [167,168] (ii) Leptin promoted growth and progression of breast cancers, especially in K303R-associated breast cancers [169,170]. (iii) Leptin sustained breast cancer stem cell phenotype [170,171] (iv) Leptin induced epithelial-to-mesenchymal transition in normal breast cells, which may increase the risk of breast cancer [172]. (v) Leptin reduced the number of cells of G0/G1 phase and by incrementing the number of breast cancer cells with the S and G2/M phases [173]. (vi) Leptin induced the expression of Vascular Endothelial Growth Factor (VEGF) contributing to angiogenesis and cancer progression [173]. (vii) Leptin modulated extracellular vesicle biogenesis and cargo [174,175]. (viii) Leptin increased tumor aggressiveness in mammary tissues of a rat model of breast cancer driven by diet-induced obesity [176]. (ix) Leptin reduced tumor growth and numbers of lung and liver metastases in SCID (severe combined immunodeficiency)/beige mice, implanted with ObR-knockout adipose stromal/stem cells isolated from obese patients [177]. (x) Lack of leptin signaling activation was correlated with a decrease in mammary tumor development [178,179,180]. (xi) Obese mice with hyperleptinemia showed increased tumor progression and a poor response to tamoxifen compared to non-obese mice [181]. (xii) The lack of ObR reduced breast cancer growth and the recruitment of macrophages [182].	(i) Leptin serum levels have been found associated with breast cancer risk [183,184]. (ii) Leptin/Leptin receptor have been found in high-grade breast cancer and positively correlated with poor prognosis and distant metastasis [39,40,41,42,43,44]. (iii) High leptin receptor expression correlated with reduced overall survival in breast carcinoma patients, especially in basal-like subtypes cancers [171].	(i) LDFI completely reversed the breast cancer stem cell phenotype [171]. (ii) LDFI blocked leptin-induced extracellular vesicle biogenesis [174]. (iii) Leptin peptide receptor antagonist linked to nanoparticles blocked leptin induced-effects on breast cancer growth, stemness and response to therapy [170].
***Ovarian***	(i) Leptin induced cell growth and proliferation in several ovarian cancer cell lines [185,186,187,188,189]. (ii) Leptin regulated cell proliferation through the activation of PI3K/AKT pathways along with mitogen-activated protein kinase (MEK)/ERK1/2 signaling [185] (iii) Leptin induced migration and invasion through the involvement of RhoA/ROCK, PI3/AKT and JAK/STAT3 pathways; by activation of matrix metalloproteinase MMP-9 and via uPA upregulation [190,191,192,193]. (iv) Leptin stimulated cell migration by activation of matrix metalloproteinase MMP-9 [191]. (v) Leptin maintained stemness and mesenchymal phenotype contributing to tumor progression [192]. (vi) A mouse ovarian cancer ID-8 cells implanted in female C57B6 mice fed a high-energy diet (HED) compared to the regular diet (RD) and a caloric restriction diet (CRD) group displayed high circulating levels of leptin, along with a more extensive tumor volume at all the peritoneum-related organ and metastatic sites [194].	(i) Ovarian cancer patients with high leptin circulating levels experienced worse overall survival [192]. (ii) Leptin/leptin receptor are highly expressed in metastatic than in primary tumors [192]. (iii) Ovarian cancer group exhibited lower levels of leptin compared to the group with benign ovarian mass [195]. (iv) Leptin overexpression has been considered a positive prognosticator of disease-free survival along with disease-specific survival in ovarian cancer patients [196].	(i) Human ObR antagonists reversed the leptin-stimulatory effects on proliferation of ovarian cell lines impairing the crosstalk between leptin and estrogen signaling [189].
***Cervical***	(i) Leptin levels were increased in cervical cancer cells in a dose-dependent fashion and were correlated with increased protein and mRNA levels of c-myc, which is a well-known oncogene [48]. (ii) Leptin silencing decreased leptin-induced proliferation, along with reduced expression protoncogene c-myc and antiapoptotic protein bcl-2 in HeLa cell line [48].	(i) OBR expression correlated with poor outcomes in women with cervical cancer, whereas high leptin levels have been found associated with favorable outcomes [197,198]. (ii) Leptin serum levels in cervical carcinoma patients have been found similar to the control group [199]. (iii) Leptin levels in patients with cervical carcinoma positively correlated with a higher grade of cervix carcinoma [48].	N/A
***Endometrial***	(i) Leptin induced endometrial cancer cell proliferation [200,201,202]. (ii) Leptin induced cancer cell migration and invasion [200,203]. (iii) Leptin decreased the G0/G1-phase cell population and increased S-phase cell population [202]. (iv) Leptin protected Ishikawa and HEC-1A cells from apoptosis [204].	(i) Leptin circulating levels positively correlated with endometrial cancer’s risk [205,206]. (ii) Endometrial cancer tissues displayed higher circulating levels of leptin/leptin receptor in comparison with normal endometrial tissues [55]. (iii) Higher leptin levels have been found associated with lymph vessel involvement and poorly neoplastic differentiation [207].	Leptin-derived peptide restored chemotherapy sensitivity [200].
